# Metagenomic Analysis of Gut Microbiome Across Developmental Stage of Asian Corn Borer (*Ostrinia furnacalis*)

**DOI:** 10.3390/insects17050495

**Published:** 2026-05-13

**Authors:** Mengfan Tao, Jianzhen Zhang, Yunhe Fan

**Affiliations:** 1 School of Life Science, Shanxi University, Taiyuan 030006, China; taomf615@163.com; 2 Shanxi Key Laboratory of Nucleic Acid Biopesticides, School of Synthetic Biology, Shanxi University, Taiyuan 030006, China; 3 Shanxi Bethune Hospital, Shanxi Academy of Medical Sciences, Third Hospital of Shanxi Medical University, Tongji Shanxi Hospital, Taiyuan 030032, China

**Keywords:** *Ostrinia furnacalis*, metagenomics, developmental stage, gut microbiome

## Abstract

*Ostrinia furnacalis* is one of the most important agricultural pests in Asia. Previous studies utilizing 16S rRNA sequencing have established a foundational understanding of the taxonomic composition of its gut microbiota; however, the dynamic functional transitions across the host’s entire life cycle remain poorly understood. In this study, we used metagenomic sequencing to systematically characterize the gut microbiome across six groups representing different life stages and sexes of *O. furnacalis*: first-instar, third-instar, and fifth-instar larvae, pupae, and adults (both males and females). Microbial richness and evenness vary significantly across six groups representing different life stages and sexes. Species richness is highest in the first-instar larvae (L1D2), while evenness is relatively high in both first- and third-instar larvae (L1D2 and L3D2). Additionally, no sex-based differences were observed in either indicator during the adult stage. *Enterococcus mundtii* is the primary species driving community succession and rapidly achieves dominance after the third-instar stage. Co-occurrence network analysis revealed that the first-instar larval network exhibits the highest complexity, with positive correlations accounting for 96.6% of all edges. Conversely, the fifth-instar larvae exhibits the greatest proportion of negative correlation edges at 29.13%, while the pupal stage network is the most dispersive, indicating microbial reorganization during metamorphosis. Functional annotation reveals that carbohydrate and amino acid metabolism pathways are significantly enriched during the larval stage. In contrast, the pupal stage is characterized by enrichment in environmental information processing and a notable increase in polysaccharide lyases (PLs). This shift indicates that the microbiota transitioned from degrading plant polysaccharides to foraging host-derived glycans. The number of resistance genes in the first-instar larvae is significantly higher than that in all other groups representing different life stages and sexes. Collectively, this study systematically reveals the dynamic succession patterns of the gut microbiome throughout the life cycle of *O. furnacalis* and provides a theoretical foundation for the development of microbiome-based pest management strategies.

## Simple Summary

*Ostrinia furnacalis* is one of the most crucial agricultural pests in Asia. It employs a stage-specific dynamic succession strategy within its gut microbiome to adapt to the drastic physiological and dietary changes which occur during holometabolous metamorphosis. In the newly hatched stage, the species harbors a diverse microbiota featuring a highly complex co-occurrence network (dominated by 96.6% positive correlations). However, in later developmental stages, the gut microbiota becomes robustly enriched with *Enterococcus mundtii*, driving intense competitive exclusion. Microbial functions are significantly regulated by the developmental stage, exhibiting pronounced differences between the feeding larval phase and the fasting pupal phase. During the hyperphagic fifth-instar larval stage, the microbial community is primarily enriched in carbohydrate metabolism pathways for the degradation of plant polysaccharides. Conversely, in the pupal stage, the microbiota undergoes a significant functional shift toward degrading host-derived endogenous glycans via polysaccharide lyases (PLs). These findings indicate that the gut microbial remodeling of this species is intricately linked to the host’s developmental transitions and metabolic demands, providing critical insights into the co-evolutionary adaptation mechanisms of agricultural pests.

## 1. Introduction

The *Ostrinia furnacalis*, a member of the family Crambidae (Lepidoptera), is one of the most destructive agricultural pests and is widely distributed in Asia and the Western Pacific [1]. Its larvae feed on ears and are characterized by their concealed boring habit, which damages plant vascular tissue leading to disrupted nutrient transport, stem lodging, and ear rot, which typically results in an average 10–30% annual yield loss, even reaching more than 80% in severe cases [2,3]. Over the past few decades, control strategies against *O. furnacalis* have relied primarily on the application of chemical insecticides and the cultivation of genetically modified corn expressing *Bacillus thuringiensis* (Bt) toxins [4]. Bt crops control pests by specifically damaging the midgut epithelial cells of Lepidopteran insects through the expression of certain insecticidal crystal proteins, such as Cry1Ab, Cry1F, and Cry1Ie [5,6]. However, sustained selection pressure from single-target application has led to the evolution of Bt resistance in field populations. For instance, long-term susceptibility monitoring programs and laboratory selection studies have revealed that certain populations of *O. furnacalis* in China exhibit significantly reduced susceptibility or possess the genetic potential to develop high levels of resistance to Cry1Ab and Cry1F [7]. In addition, the metabolic resistance of pests to traditional chemical agents (such as macrolides and diamides) is also increasing [8,9]. Therefore, a deeper understanding of the resistance mechanism is crucial for developing novel prevention and control strategies based on the pest’s fundamental biological characteristics.

Recently, the insect gut microbiome has been recognized as the host’s “second genome” which plays a crucial role in the host’s adaptive evolution. The insect’s gut serves not only as a site for food digestion and absorption but also as a complex microecological system harboring bacteria, fungi, and viruses [10,11,12]. The gut microbes are involved in critical functions, including nutrient digestion and absorption (including lignocellulose degradation and essential amino acid synthesis), immune defense, insecticide resistance, and adaptation to environmental stressors such as extreme temperatures and plant secondary metabolites [10,13,14]. Compared with mammals or other insect orders, the actively feeding larvae of many Lepidoptera possess a unique and extreme alkalinity in their intestinal environment (pH 8.0–12.0). This extreme pH environment is mainly considered an adaptation to a plant-based diet rich in secondary metabolites such as tannins [15,16,17]. However, it is important to note that gut pH and the composition of the microbiota are highly dynamic and can vary significantly depending on the host taxon, developmental stage, and specific dietary composition [18]. Despite this variation, this stringent environment frequently acts as a strong selective barrier, generally resulting in the abundance and diversity of the intestinal microbiota of Lepidopteran larvae being lower than that of other insects [10,19]. However, such an environment does not diminish the functional significance of the resident microbes. After long-term coevolution, specific alkalitolerant bacterial groups have successfully colonized the gut and acquired irreplaceable metabolic functions [17,20]. These alkalitolerant symbiotic bacteria play an important role in participating in host nutrition, for instance by secreting the carbohydrate-active enzymes that the host lacks, thereby assisting in the degradation of recalcitrant plant cell wall polysaccharides and enhancing energy acquisition efficiency [21,22].

The life cycle of holometabolous insects comprises four distinct development stages, i.e., egg, larva, pupa, and adult, accompanied by dramatic changes in morphology, physiology, and life habits [23]. Such drastic physiological remodeling during development is inevitably accompanied by the reconstruction of the intestinal microenvironment, thereby driving the succession of the microbial community [18]. Previous studies have largely relied on 16S rRNA amplicon sequencing. Although these approaches have revealed shifts in microbial composition, systematic analyses of the dynamic succession of microbial functions are still unclear, particularly across the critical transition from larval feeding through pupal fasting to adult refeeding. Gender represents another core variable influencing insect physiology, in which male and female adults differ significantly in motility, nutritional requirements, and immune responses [24,25]. Although sex differences are well recognized in insect physiology, their impact on the gut microbiome is rarely reported, especially at the functional genome level (such as the distribution of antibiotic resistance genes), and whether sexual dimorphism exists is unclear. In this study, we performed metagenomic sequencing on intestinal samples collected from six groups representing different life stages and sexes of *O. furnacalis*: 1st, 3rd, and 5th instar larvae, pupae, and female and male adults. Our objective was to construct a comprehensive functional map of the gut microbiota across the pest’s entire life cycle and to elucidate how metamorphosis, development, and sexual differentiation shape the symbiotic microbial community. The findings are expected to provide new insights into host-microbe coevolution and to identify molecular targets for screening functional microorganisms with the potential for biocontrol applications.

## 2. Materials and Methods

### 2.1. Sample Collection

The *O. furnacalis* used in this experiment was presented by Professor Wang Zhenying from the Chinese Academy of Agricultural Sciences in September 2012. It has been continuously raised by the Laboratory of Insect Physiology, Biochemistry, and Molecular Biology of China Agricultural University. The Asian corn borer used in the experiment was raised in an intelligent artificial climate chamber (RXZ type, Ningbo Jiangnan Instrument Co., Ltd., Ningbo, China), with a photoperiod of 16L: 8D, a temperature of 28 ± 1 °C, and a relative humidity of about 60%. The artificial diet formula for the *O. furnacalis* is soybean meal, rice flour, agar, and water. Six groups of *O. furnacalis* individuals at different developmental stages and sexes were randomly selected from laboratory-reared populations: first instar (L1D2), third instar (L3D2), fifth instar larvae (L5D2), pupae, female adults (adult_F), and male adults (adult_M), with six biological repetitions per group. To ensure that sufficient microbial DNA is obtained through sequencing methods for large-scale sequencing, and considering the significant differences in individual sizes at different developmental stages, the sample size for a biological replicate is 0.3 g. The surface of each sample was disinfected with 75% ethanol, followed by two rinses in sterile distilled water. Dissecting instruments were sterilized by autoclaving at 121 °C for 30 min prior to use. Under sterile conditions, the intestinal tracts of larvae, pupae, and adults were dissected, transferred into sterile 1.5 mL microcentrifuge tubes, and immediately stored at −80 °C.

### 2.2. Metagenome DNA Extraction and Shotgun Sequencing

Total microbial genomic DNA was extracted from intestinal samples using the OMEGA Mag-Bind Soil DNA Kit (M5635-02) (Omega Bio-Tek, Norcross, GA, USA) according to the manufacturer’s instructions. The extracted DNA was stored at −20 °C prior to analysis. DNA concentration and purity were assessed using a Qubit™ 4 Fluorometer, (equipped with WiFi: Q33238 (Qubit™ Assay Tubes: Q32856; Qubit™ 1× dsDNA HS Assay Kit: Q33231; Invitrogen, Carlsbad, CA, USA) and DNA integrity was evaluated by agarose gel electrophoresis. Metagenomic shotgun sequencing libraries were constructed with an insert size of ~400 bp by using Illumina TruSeq Nano DNA LT Library Preparation Kit (Illumina, San Diego, CA, USA). Sequencing was performed on the Illumina NovaSeq platform (Illumina, San Diego, CA, USA) with the PE150 strategy at Personal Biotechnology Co., Ltd. (Shanghai, China).

### 2.3. Metagenomics Analysis

A total of 36 samples were sequenced by Illumina Miseq PE300 and obtained 1, 682, 902, 714 pairs of reads (Table S1). Raw sequencing reads were processed to obtain quality-filtered reads for further analysis. First, this involved adapter removal and the trimming of low-quality reads using fastp (v 0.23.2) [26]. Subsequently, reads were aligned to the host genome of *O. furnacalis* using Minimap2 (version 2.24-r1122). Thirdly, reads were aligned to the host genome of *O. furnacalis* using Minimap2 (v2.24-r1122) to remove host contamination [27]. Then, MEGAHIT (version 1.1.2) software was used to concatenate and assemble the valid reads sequences of each sample, with contigs of no less than 300 bp length retained by default [28]. Prodigal (version 2.6) software (https://github.com/hyattpd/Prodigal/, accessed on 11 November 2025) was used to identify open reading frames (ORFs) and predict the coding regions to obtain corresponding gene and protein sequence files [29]. At the same time, the cluster module of MMseq (version 13.45111) software was used to remove redundancy and obtain non redundant gene and protein sets. To ensure that the presence of dietary or residual host DNA did not bias the results, any reads assigned to Metazoa or Viridiplantae were strictly excluded from the dataset prior to any quantitative analysis. To determine the overall microbial community composition, taxonomical classifications were performed directly on the obtained quality-filtered reads, and taxonomical classifications of metagenomic sequencing reads from each sample were performed using Kraken2 (v2.0.8-beta) [30], which included atabase archaeal and bacterial genomes from the GTDB database (2024), viral sequences from the RVDB database (2024), and eukaryotic microbial sequences from the NCBI-nt database (2024). Integrating these highly curated, domain-specific databases alongside NCBI-nt allowed us to optimize the specificity and accuracy of our taxonomic assignment. Reads assigned to metazoans or Viridiplantae were removed for compositional analysis. In parallel, to investigate the functional potential of the microbiome, a contig-based assembly strategy was employed. To strictly ensure the accuracy of downstream functional annotations by removing any residual host or dietary sequences, the nonredundant contigs were further filtered by aligning them against the NCBI-nt database by mmseqs2 with “taxonomy” mode, and contigs assigned to Viridiplantae or Metazoa were dropped in the following functional analysis. Prodigal (V2.6.3) [29] was used to predict the genes in the contigs. CDS sequences of all samples were clustered by mmseqs2 with “easy-cluster” mode, setting the protein sequence identity threshold to 0.95 and covered residues of the shorter contig to 90%. To estimate gene abundance, high-quality reads from each sample were back to the non-redundant gene sequences using Minimap2. Read counts per gene were calculated, and Reads Count (RC) values were used as a proxy for abundance [31] for each gene. The functionality of the non-redundant genes was obtained by annotation using mmseqs2 with the “search” mode against the protein databases of KEGG and CAZy databases and using Diamond (v2.0.15) against CARD respectively. To ensure high-confidence functional predictions, stringent filtering criteria were applied to the alignment results. Specifically, alignments were retained only if they met the following thresholds: an E-value < [1 × 10^−5^], a minimum amino acid sequence identity of [30]% and a query coverage of at least [50]%. For multiple hits assigned to a single gene, the best match was selected based on the lowest E-value. If E-values were identical, the hit with the highest bit-score was chosen as the definitive annotation. The parallel use of these specialized, highly curated databases alongside the comprehensive NCBI-nt database was implemented to maximize both the sensitivity and taxonomic resolution of our profiling while minimizing potential misclassifications.

### 2.4. Statistical Analyses

To assess the normality of the data prior to microbial diversity analysis, the Shapiro–Wilk test was first applied. For comparisons of within-sample (alpha) data, Student’s *t*-test was used for normally distributed data when comparing two groups, while one-way analysis of variance (ANOVA) followed by Tukey’s post hoc test were employed for multiple group comparisons. To identify differentially abundant taxa and functional features among groups, Random Forest analysis was conducted based on the taxonomic and functional profiles of non-redundant genes using default parameters [32]. Beta diversity was assessed to evaluate compositional and functional differences in microbial communities across samples using Bray–Curtis dissimilarity [33], and was visualized via principal coordinate analysis (PCoA). Co-occurrence network analysis was performed using R 3.2.6 and Gephi v.0.9.2 software (*p* < 0.05, |R| > 0.6) [34]. Statistical significance was defined as * *p* < 0.05, ** *p* < 0.01, and *** *p* < 0.001.

## 3. Results

### 3.1. Composition and Abundance Analysis of Gut Microbiota

**Figure 1:**
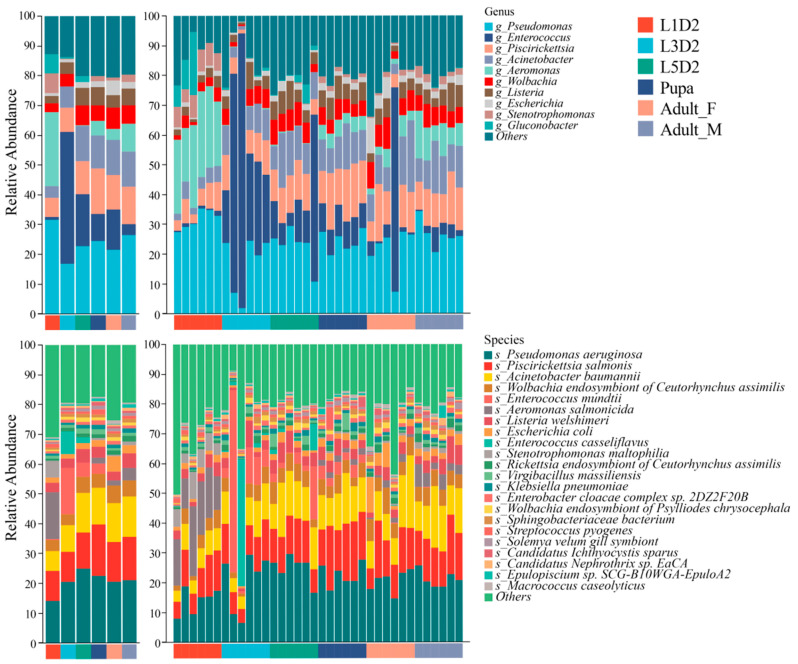
The composition of gut microbiota at genus and species level in *Ostrinia furnacalis*. Taxa with a relative abundance of less than [1%] across all samples, along with unclassified sequences, were aggregated and designated as ‘Others’.

To visualize the distribution of dominant bacteria across groups, relative abundance plots of gut microbiota in *O. furnacalis* were generated at the genus and species level. Metagenomic sequencing results showed that the dominant bacterial genera in the intestinal microbiota of *O. furnacalis* were *Pseudomonas* (21.21% ± 6.276%), *Enterococcus* (7.705% ± 15.72%), *Piscirickettsia* (12.16% ± 4.409%), *Acinetobacter* (12.16% ± 4.409%), *Aeromonas* (6.212% ± 8.852%), *Wolbachia* (5.814% ± 2.248%), *Listeria* (4.390% ± 1.636%), *Escherichia* (5.814% ± 2.248%), and *Stenotrophomonas* (1.889% ± 2.235%). At the species level, the dominant bacterial species were *Pseudomonas aeruginosa* (19.400% ± 4.631%), *Piscirickettsia salmonis* (12.046% ± 3.230%), *Acinetobacter baumannii* (11.550% ± 2.741%), *Wolbachia endosymbiont of Ceutorhynchus assimilis* (4.472% ± 1.249%), *Enterococcus mundtii* (3.377% ± 5.641%), *Aeromonas salmonicida* (3.371% ± 5.743%), *Listeria welshimeri* (3.484% ± 1.211%), and *Escherichia coli* (3.831% ± 3.732%) (Figure 1 and Table S2).

We further analyzed the differences in the abundance of these genera and species among the groups. At the genus level, the relative abundances of *Pseudomonas*, *Enterococcus*, *Aeromonas*, and *Wolbachia* were relatively high during the larval stage (L1D2, L3D2, and L5D2 groups) and were significantly higher than in the pupal stage and adult stage (adult_F and adult_M groups). In addition, the relative abundance of *Stenotrophomonas* gradually increased with the development of *O. furnacalis*, with the abundance in the adult stage being significantly higher than that in the pupal stage and early-instar larval stages. At the species level, the relative abundance of *Pseudomonas aeruginosa*, *Enterococcus mundtii*, and *Aeromonas salmonicida* was relatively high during the larval stage (L1D2, L3D2, and L5D2 groups) and was significantly higher than those in the pupal and adult stages (adult_F and adult_M groups). Notably, no sex-based differences in the relative abundance of bacterial communities were observed among adults (Figure 1).

### 3.2. Analysis of the Diversity and Differential Bacterial Communities in the Gut

**Figure 2:**
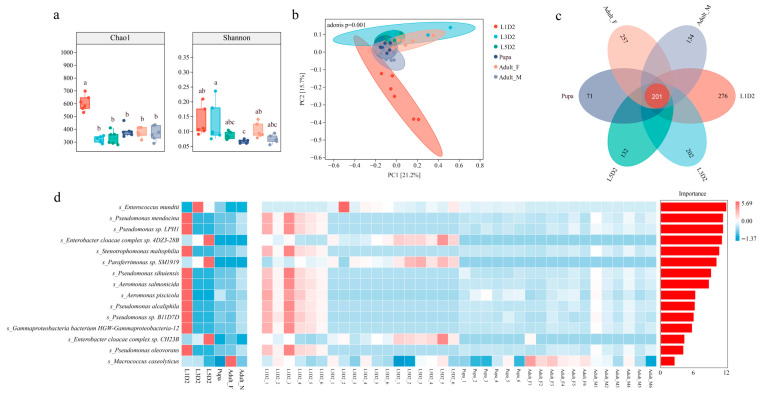
Analysis of diversity and differential bacterial communities in gut of *O. furnacalis*. (**a**) Analysis of gut microbial alpha diversity in *Ostrinia furnacalis*. (**b**) Analysis of gut microbial beta diversity in *Ostrinia furnacalis*. (**c**) Petal diagram of number of genes in the microbiome of *Ostrinia furnacalis*. (**d**) Heat map of Random Forest analysis of gut microbiota of *Ostrinia furnacalis*. Different lowercase letters above the Figure a denote statistically significant differences among the groups (*p* < 0.05), whereas no significant differences exist between groups sharing the same letter.

To evaluate changes in gut microbial diversity in *O. furnacalis* at different developmental stages and sexes, we analyzed Chao1 and Shannon indices (Figure 2a). The results show that there are significant differences in the richness and evenness of the microbial communities. The Chao1 index indicated that microbial richness was highest in the L1D2 group and was significantly higher than that in all other developmental stages and sexes. In contrast, no significant difference was observed between the L3D2 and adult stages. The Shannon index indicated that microbial evenness in the L1D2 and L3D2 stages remained high and was significantly higher than that during the pupal stage. Evenness then rebounded significantly in the adult stage. However, no sex-based differences in microbial richness or evenness were observed in adults. Principal coordinate analysis (PCoA) based on beta diversity metrics revealed distinct shifts in the intestinal microbial community structure across different developmental stages and sexes of *O. furnacalis* (Figure 2b). PCoA revealed distinct clustering according to developmental stages and sexes. Specifically, samples from the L1D2 stage were clearly separated from all subsequent developmental stages and sexes along the PCoA1 and PCoA2 axes, indicating that they possessed a unique microbial community structure. Permutational multivariate analysis of variance (PERMANOVA; Adonis) further confirmed these observations, revealing highly significant differences in community structure across developmental stages and sexes (*p* < 0.001).

A flower plot was constructed to visualize the core and unique microbial taxa across the entire life cycle of *O. furnacalis* (Figure 2c). A total of 201 core microbial taxa were shared across all developmental stages and sexes from first-instar larvae to adults. The L1D2 stage has the most endemic taxa (276), whereas the pupal stage exhibited the fewest endemic taxa (71). This pattern was consistent with the alpha diversity trends observed across developmental stages and sexes. To identify key species driving microbial community succession across developmental stages and sexes we performed Forest-based feature importance analysis (Figure 2d). The results showed that *Enterococcus mundtii* was the most critical species to distinguish each stage. Its abundance was minimal in the L1D2 stage but increased sharply thereafter, becoming the dominant species in the third-instar larvae, fifth-instar larvae, pupal, and adult stages.

### 3.3. Functional Analysis Based on the KEGG and CAZy Database

**Figure 3:**
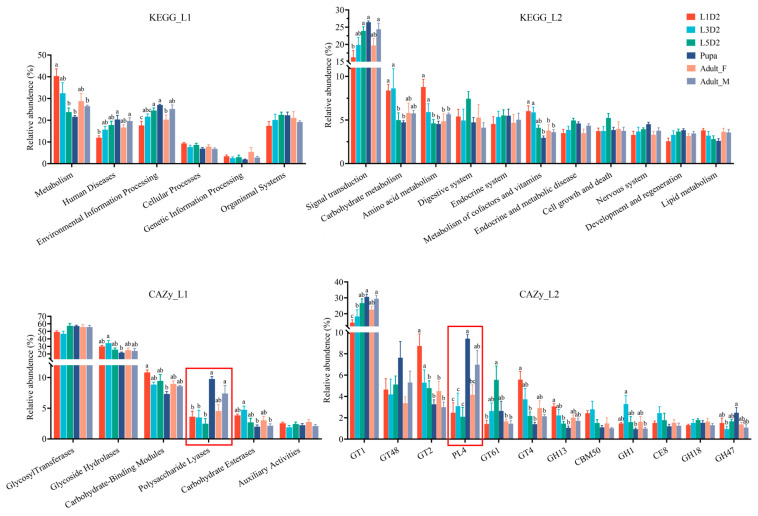
Analysis of the differences in gut microbiome function at level 1 and level 2 based on the KEGG database. Different lowercase letters above the bars denote statistically significant differences among the groups (*p* < 0.05), whereas no significant differences exist between groups sharing the same letter. Red boxes are used to highlight the functional categories discussed in detail in the main text.

Based on the functional annotation (Figure 3), at KEGG pathway level 1 metabolism was the dominant category. Its relative abundance was highest in the L1D2 group and was significantly higher than those in the L5D2, pupal, and adult_M groups. This abundance showed a decreasing trend across developmental stages and sexes. In contrast, Environmental Information Processing was significantly enriched in the L5D2 and pupal groups, with relative abundances significantly higher than those in the L1D2 and adult_F groups. At level 2, the relative abundance of Signal transduction was significantly lower in the L1D2 group than that in the L5D2, pupal, and adult_M groups. The abundance of Carbohydrate metabolism and Amino acid metabolism was significantly higher in the L1D2 and L3D2 groups than in the pupal group. Additionally, the abundance of the metabolism of cofactors and vitamins was significantly higher in the L1D2 and L3D2 groups than in the pupal, adult_F, and adult_M groups (Figure 3). Further multi-level analysis (Supplementary Table S3) revealed that broad Level 1 and Level 2 categories were primarily driven by specific Level 3 functional modules, prominently including the phosphatidylinositol and sphingolipid signaling systems, as well as conserved homologs related to insulin resistance and apoptosis.

Based on CAZy functional annotation, at the CAZy class level, the abundance of Glycoside Hydrolases (GHs) was highest in the L3D2 group and was significantly higher than that in the pupal group. Similarly, the abundance of Carbohydrate-Binding Modules (GBMs) was highest in the L1D2 group and was significantly higher than that of in the pupal group. The abundance of Polysaccharide lyases (PLs) was low across all the larval stages but increased significantly in the pupal group and remained at a relatively high level in the adult_F and adult_M groups. The abundance of Carbohydrate Esterases (CEs) was highest in the L3D2 group and was significantly higher than that in the pupal and adult_M groups. At family level, GlycosylTransferase family 1 (GT1) was the dominant family, and its abundance increases significantly with the development of *O. furnacalis*, peaking in the pupal stage and in male adults. In contrast, the abundance of GT2 and GT4 were significantly enriched in the L1D2 group but decreased markedly in the pupal group. PL4 abundance increased significantly in the pupal group, reaching levels substantially higher than those in all larval stages. GH1 abundance was relatively high in the L3D2 group, while GH47 abundance peaked in the pupal stage. These patterns indicate significant stage-specific differences in the abundance of these CAZy families across development (Figure 3).

### 3.4. Analysis of Antibiotic Resistance Genes

**Figure 4:**
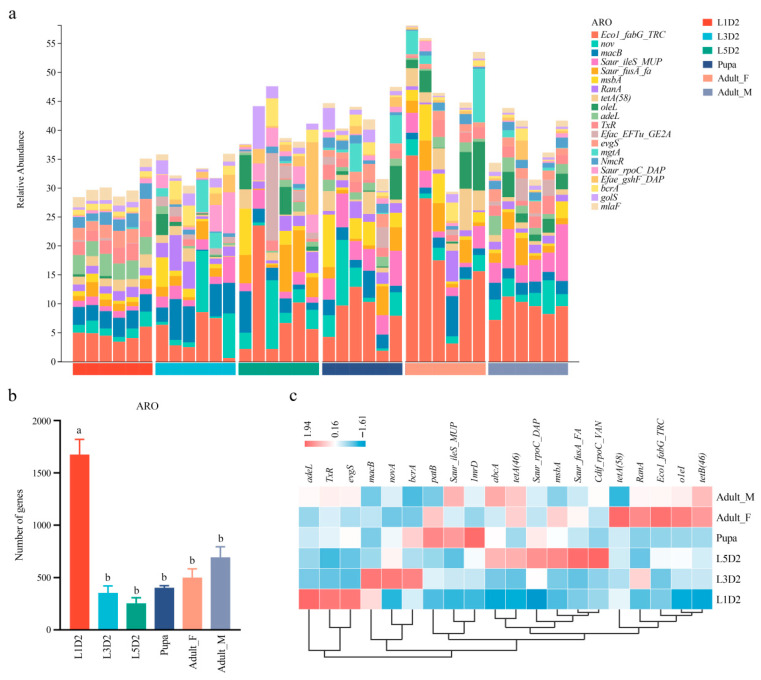
Analysis of antibiotic resistance genes. (**a**) Bar chart of abundance of different antimicrobial resistance ontologies (AROs) in each sample. (**b**) Analysis of resistance genes from different groups. (**c**) ARO abundance clustering heat map. Note: In Figure (**b**), different lowercase letters indicate a statistically significant difference between the first group and all other groups (*p* < 0.05), whereas there are no significant differences among the remaining groups sharing the letter “b”.

Based on the relative abundance of resistance genes (the original relative abundance data were magnified by 10^6^ times), the abundance of AROs was calculated, and AROs with the top 20 abundances were screened for data analysis. (Figure 4a). Across all samples, the five most abundant AROs were *Ecol_fabG_TRC*, *novA*, *macB*, *Saur_ileS_MUP*, and *Saur_fusA_FA*. Intergroup comparison revealed that the L1D2 group harbored a higher number of ARO than all other groups (Figure 4b). The ARO abundance clustering heatmap further revealed distinct clustering patterns across developmental stages and sexes, indicating stage-specific ARO profiles. Specific AROs exhibited stage-specific enrichment patterns: The L1D2 group showed higher abundances of *adeL*, *TxR*, and *evgS*; The L3D2 group was characterized by higher abundances of macB, novA, and bcrA; The L5D2 group displayed elevated abundances of *Saur_rpoC_DAP*, *msbA*, *Saur_fusA_FA*, and *Cdif_rpoC_VAN*; The pupal group exhibited higher abundances of *patB*, *Saur_ileS_MUP*, and *lmrD* compared to other stages. Notably, although no significant sex-based differences in microbial composition were observed between adult males and females (adult_F and adult_M groups), distinct differences were detected at the functional gene level, particularly in ARO profiles. Specifically, *tetA (58)*, *RanA*, *Eco1_fabG_TRC*, *oleI*, and *tetB (46)* were enriched in the adult_F group, whereas *Saur_ileS_MUP*, *abcA*, and *tetB (46)* showed higher abundances in the adult_M group (Figure 4c).

### 3.5. Co-Occurrence Network of Core Bacteria

**Figure 5:**
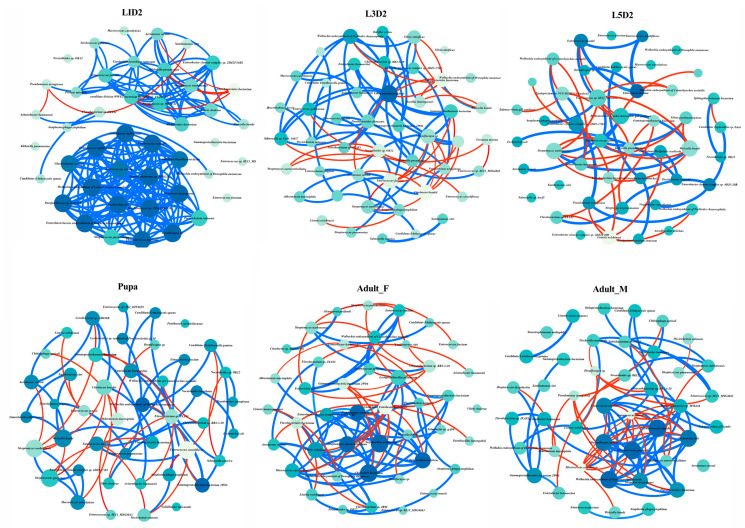
Co-occurrence networks of the 44 most abundant genera from different groups. The blue line in the figure represents a positive correlation, while the red line represents a negative correlation. The darker the color of the dot, the greater its importance; conversely, the lighter the color, the less important it is.

Co-occurrence network analysis was performed on the top 1% most abundant bacterial species across different developmental stages and sexes (Figure 5 and Table 1). The microbial network of the first-instar larvae (L1D2) exhibited the highest complexity and density, as indicated by the greatest total number of edges, average degree, and network density among all groups. Notably, it also had the highest positive correlations (96.6%). Additionally, its network diameter and average path length were the smallest among all groups, further indicating a tightly connected network structure. From the third-instar (L3D2) to the fifth-instar (L5D2) and the pupal stages, network parameters showed a general declining trend. Specifically: The L5D2 group exhibited the lowest total number of edges, average degree, and network density, while simultaneously showing the highest proportion of negative correlations among all groups (29.13%): The pupal stage was characterized by the lowest average clustering coefficient, the largest average path length, and a network diameter comparable to that of the L5D2 group. In the adult stage, the total number of edges rebounded to 128 in the female (Adult_F) network and 129 in the male (Adult_M) network. The proportions of positive and negative correlations were similar between the two adult groups. Compared with the pupal stage, both average degree and average clustering coefficient increased in adults (Table 1 and Figure 5).

## 4. Discussion

The insect gut microbiome is a highly dynamic ecosystem that is intimately linked to host physiology, diet, and development. In this study, we performed a comprehensive metagenomic analysis of the gut microbiome of *O. furnacalis* throughout its life cycle. Although there were no negative controls in this study, we carried out strict sterilization and strict data control during the sampling process. In the first-instar larvae (L1D2), we observed remarkably high alpha diversity (as measured by Chao1 and Shannon indices) and network complexity. This suggests that newly hatched larvae passively acquire a wide range of environmental and food-associated microorganisms [19,35]. This ecological pattern is highly consistent across other lepidopteran insects. For example, studies on the life cycle of *Grapholita molesta* and *Spodoptera frugiperda* showed that gut microbiota abundance peaked during the initial hatching stage, followed by a dramatic succession pattern as larvae aged [36,37]. At this early developmental stage and sex the physicochemical barriers of the insect gut (such as the highly alkaline environment and the production of antimicrobial peptides) have not yet been fully established. This facilitates transient colonization by *Acinetobacter* and *Pseudomonas* [38,39]. However, as the larvae mature (L3D2 and L5D2 stages), the intestinal environment exerts intense selective pressure. Under this selective pressure, *Enterococcus mundtii* rapidly established dominance, leading to a sharp decline in overall diversity. This taxonomic shift is highly consistent with previous 16S rRNA-based surveys of *O. furnacalis* which highlight the transition toward *Enterococcus mundtii* dominance in mature larvae [40]. Our metagenomic data not only confirm these taxonomic patterns but also provide a genomic explanation for this dominance, such as the presence of specific bacteriocin encoding genes that facilitate competitive exclusion [21]. This bacterium is common and widely distributed in lepidopteran insects. A similar community reorganization has been observed in *Spodoptera littoralis*, and its underlying mechanism has been elucidated at the molecular level: *Enterococcus mundtii* forms a robust biofilm on the intestinal epithelium and secretes bacteriocins, thereby outcompeting and excluding opportunistic environmental bacteria [41,42]. Furthermore, studies on *Galleria mellonella* have corroborated these findings, demonstrating that the competitive exclusion and chemical inhibition capabilities of *E. mundtii*, together with the host immune system, drive the simplification and stabilization of the gut microbiota in later developmental stages and sexes [38,40].

Co-occurrence network analysis of the gut microbiota across different developmental stages and sexes of *O. furnacalis* clearly demonstrated competitive exclusion among microbial communities. The L1D2 network was the densest and was predominantly characterized by positive correlations (96.6%). In contrast, the L5D2 network exhibited the lowest density and the highest proportion of negative antagonistic edges (29.13%). High proportions of negative correlations in microbial networks are often interpreted as indicators of intense resource competition or chemical antagonism [42,43]. In the context of our study, this suggests that dominant bacteria, such as *Enterococcus mundtii*, actively monopolize ecological niches and suppress competing taxa through spatial occupation and the secretion of antimicrobial compounds. This competitive pressure likely drives the proliferation of antagonistic interactions within the community network. In addition, the pupal stage shows the lowest clustering coefficient and the largest network diameter. Previous studies have shown that the extensive intestinal remodeling during the pupal stage not only eliminates most of the resident microbiota but also completely disrupts the topological structure of the microbial network through physical barrier restructuring and heightened immune responses [38]. These findings suggest that metamorphosis profoundly reshapes the microbial community and its interspecific interactions. Therefore, the network fragmentation observed in the pupal stage in our study reflects the profound physiological restructuring that holometabolous insects undergo during metamorphosis and its impact on gut microbial community structure.

Functional annotation based on the KEGG and CAZy databases revealed the relationship with the shifting metabolic demands of the host across development. The significant enrichment of carbohydrate and amino acid metabolic pathways, coupled with the high abundance of glycoside hydrolases (GHs) and carbohydrate-binding modules (CBMs) during the actively feeding larval stage, highlights the mutualistic role of the microbiota in lignocellulose degradation and the extraction of nutrients from maize tissues [10,44]. This finding is consistent with recent metagenomic studies on the gut microbiota of polyphagous lepidopteran larvae including *Helicoverpa armigera* and *Spodoptera frugiperda*. Studies have also demonstrated that during the rapid growth phase of larvae, the symbiotic microbiota upregulates the expression of specific GH family enzymes, thereby assisting the host in degrading complex plant polysaccharides and breaching the physical barriers of plant cell walls [45,46]. In contrast, the pupal stage is a period of physiological fasting, during which the insect ceases the ingestion of exogenous plant polysaccharides, and its gut undergoes extensive epithelial cell shedding and remodeling. Our data show that the microbiota in the pupal stage is enriched in environmental information processing functions, indicating that bacterial ABC transporters are heavily activated in response to nutrient starvation, leading to a microbial stress response [10]. Functionally, polysaccharide lyases (PLs) typically target uronic acid-containing polysaccharides, which are core structural components of host-derived mucins and glycosaminoglycans [47]. Thus, the shift from GHs enrichment in the larval stage to PLs enrichment in the pupal stage represents not only a microbial stress response to nutrient scarcity but also provides direct metagenomic evidence for glycan foraging [48]. This metabolic shift has been well documented across diverse host–microbe symbiotic associations. For instance, in models of nutrient deprivation, when dietary polysaccharides are limited, the gut microbiota upregulates polysaccharide lyases (PLs) that target host mucins as an alternative carbon sources [49]. The functional shift observed in *O. furnacalis* during the pupation not only corroborates this general ecological principle but also provides additional insights. Specifically, during the intestinal remodeling that characterizes holometabolous metamorphosis, the symbiotic microbiota appears to degrade host-derived endogenous polysaccharides and may indirectly contribute to the clearance of sloughed epithelial cells and nutrient recycling [10,50].

In this study, we conducted a comprehensive analysis of antibiotic resistance genes throughout the development of *O. furnacalis*. Although the clinical significance of antibiotics is frequently discussed in the context of humans and livestock, the resistomes of agricultural pests primarily reflect ecological adaptation to environment pressures and competitive interactions among microorganisms [51]. This notion has been corroborated by recent studies on the intestinal resistance genomes of important agricultural insects such as *Bombyx mori* and *Apis mellifera* [52,53]. The distribution of gut AROs in these insects is primarily driven by microhabitat selection rather than direct antibiotic exposure. Our results show that the L1D2 stage harbored the highest abundance of AROs including *Ecol_fabG_TRC*, and *macB*. Previous studies have shown that the newly hatched larvae of lepidopteran pests such as *Plutella xylostella* and *Spodoptera frugiperda* acquired exogenous AROs from environmental sources. By feeding on the diverse microbiota that colonize the plant foliage, these larvae accumulate an initial repertoire of resistance genes [46,54]. These AROs were most likely introduced via soil- and plant-associated bacteria [55]. As the core microbiome stabilizes and bacterial diversity declines during later developmental stages and sexes, ARO diversity correspondingly decreases. Interestingly, no significant sex-based differences were observed in the microbial composition of the adults, but the clustering of AROs did show sex-based differences (e.g., *tetA* and *oleI* in females versus *abcA* in males). This sexual dimorphism in ARO profiles may stem from subtle microhabitat differences between sexes or from the selective transmission of ARO-carrying taxa to offspring by females during oviposition, a potential strategy to enhance egg protection. For instance, studies on *Bactrocera dorsalis* and *Laodelphax striatellus* have shown that resistance and detoxification genes are enriched in the gut tract and reproductive tract of adult females. Further multi-omics analyses confirmed that females can transmit symbiotic bacteria harboring these genes to the egg surface or interior (inside the eggs) thereby conferring an early competitive advantage to their offspring [56,57]. Collectively, the enrichment of specific AROs in female adults of *O. furnacalis* observed in this study may represent a transgenerational protection strategy. This hypothesis warrants further validation through approaches such as transcriptomics or fluorescence in situ hybridization in future studies.

## 5. Conclusions

**Figure 6:**
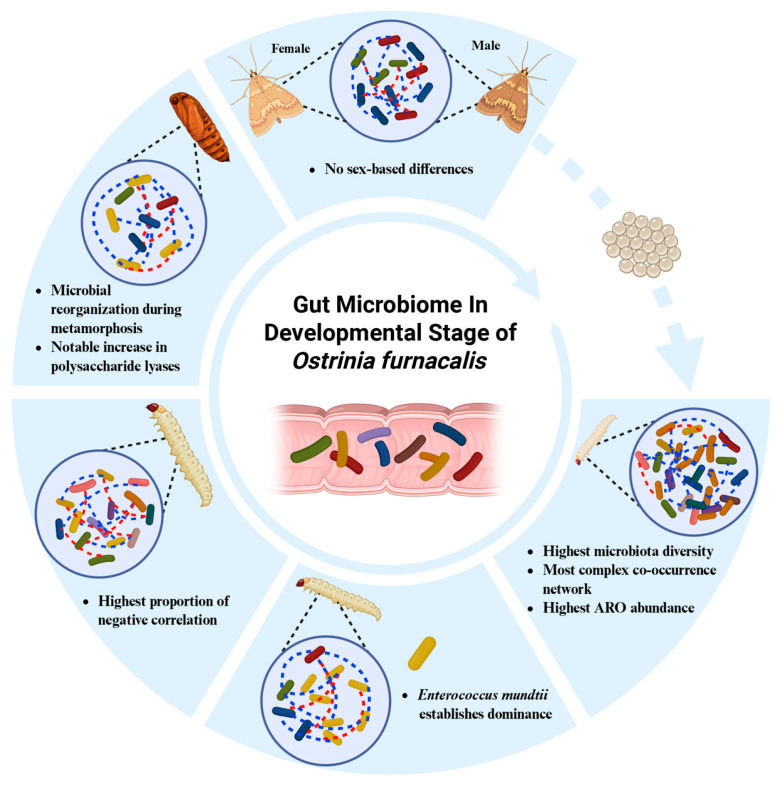
Model illustrating gut microbiome in developmental stage of *Ostrinia furnacalis*. The blue and red dotted lines in the figure correspond to the microbial co-line network, and different colored bacteria represent the diversity of intestinal species.

In this study, we systematically characterized the gut microbiome of *O. furnacalis* across its entire life cycle from first instar-larvae to adults through metagenomic sequencing, as shown in our model. Our results revealed that the composition, diversity, and function of the gut microbial community underwent significant stage-specific succession during host development. In the first instar-larval stage, gut microbiota diversity was highest, and the co-occurrence network was most complex, with positive correlations predominating. This pattern reflects the passive acquisition of environmental microorganisms by newly hatched larvae. As the larvae matured, intestinal selection pressure intensified. *Enterococcus mundtii* rapidly established dominance (position), diversity declined sharply, and the proportion of negative correlations in the network increased significantly, indicating intensified competitive exclusion among bacterial groups. In the pupal stage, bacterial community diversity was lowest, and the network structure was most fragmented. Functionally, the microbiota shifted from carbohydrate and amino acid metabolism during the larval stage toward an enrichment of polysaccharide lyases (PLs) in the pupal stage, suggesting a potential metabolic transition toward the degradation of host-derived glycans in response to nutrient scarcity. However, the specific roles of the microbiota in host-derived glycan foraging remain a hypothesis that requires further empirical validation via metatranscriptomics or biochemical assays. Collectively, these findings provide an important foundation for a deeper understanding of the symbiotic relationship between pests and their gut microbiota, as well as for the development of microbiome-based strategies for pest management (Figure 6).

## Figures and Tables

**Table 1:** Network indices of the co-occurrence network of core bacteria.

Network Indices	L1D2	L3D2	L5D2	Pupa	Adult_F	Adult_M
Total nodes	44	44	44	44	44	44
Total edges	235	117	102	104	128	129
Positive edges (%)	96.6%	77.78%	70.87%	75.96%	77.34%	77.52%
Negative edges (%)	3.4%	22.22%	29.13%	24.04%	22.66%	22.48%
Average degree	10.682	4.979	2.304	2.727	3.818	5.864
Network diameter	3	8	10	10	9	9
Average path length	1.286	3.577	3.447	4.525	2.582	3.602
Density	0.248	0.108	0.051	0.063	0.089	0.136
Average clustering coefficient	0.885	0.642	0.445	0.363	0.653	0.775

## Data Availability

Metagenome data have been uploaded to the European Nucleotide Archivedatabase (http://www.ebi.ac.uk/ena, accessed on 8 April 2026). The data entry number is PRJEB111189.

## Supplementary Materials

The following supporting information can be downloaded at: https://www.mdpi.com/article/10.3390/insects17050495/s1, Table S1: Summary of sequence statistics for the Illumina MiSeq runs for all samples. Table S2: Table of gut microbial genus and species level abundances of *O. furnacalis*. Table S3: Analysis of the differences of gut microbiome function at level 3 based on the KEGG database.
