# Supplementary material for: Metagenomic Analysis of Gut Microbiome Across Developmental Stage of Asian Corn Borer (Ostrinia furnacalis)
Source: Insects. 2026 May 13;17(5):495. doi: 10.3390/insects17050495 (PMC13206882; doi:10.3390/insects17050495)
Supplement: Supplementary file 1 [file insects-17-00495-s001.zip › insects-4279522-supplementary.pdf]

Table S1. Summary of sequence statistics for the Illumina MiSeq runs for all samples.

Sample	DNA yield	Total reads	Total valid reads	Percentage of effective sequences (%)	average sequencing depth
L1D2_1	1 μg	41388228	40914324	98.85	6 G
L1D2_2	1 μg	39046072	38626684	98.93	6 G
L1D2_3	1 μg	43795130	43331790	98.94	6 G
L1D2_4	1 μg	44526104	44050722	98.93	6 G
L1D2_5	1 μg	42108950	41664932	98.95	6 G
L1D2_6	1 μg	36008026	35643360	98.99	6 G
L3D2_1	1 μg	57202166	56479576	98.74	6 G
L3D2_2	1 μg	50153720	49507562	98.71	6 G
L3D2_3	1 μg	63659196	62867688	98.76	6 G
L3D2_4	1 μg	50555704	49915328	98.73	6 G
L3D2_5	1 μg	44762074	44213152	98.77	6 G
L3D2_6	1 μg	61080788	60265078	98.66	6 G
L5D2_1	1 μg	44512764	43941508	98.72	6 G
L5D2_2	1 μg	51791278	51068130	98.6	6 G
L5D2_3	1 μg	51649342	50975144	98.69	6 G
L5D2_4	1 μg	50231414	49663622	98.87	6 G
L5D2_5	1 μg	51390684	50771032	98.79	6 G
L5D2_6	1 μg	37726978	37241034	98.71	6 G
Pupa_1	1 μg	51242922	50698216	98.94	6 G
Pupa_2	1 μg	37807056	37400326	98.92	6 G
Pupa_3	1 μg	44336840	43874190	98.96	6 G
Pupa_4	1 μg	51502218	50891348	98.81	6 G
Pupa_5	1 μg	52734190	52183242	98.96	6 G
Pupa_6	1 μg	49364296	48845918	98.95	6 G
adult_F1	1 μg	55421206	54851766	98.97	6 G
adult_F2	1 μg	42049926	41599650	98.93	6 G
adult_F3	1 μg	50678222	50157754	98.97	6 G
adult_F4	1 μg	44698278	44190056	98.86	6 G
adult_F5	1 μg	39922404	39479778	98.89	6 G
adult_F6	1 μg	51761542	51192960	98.9	6 G
adult_M1	1 μg	44841282	44354014	98.91	6 G
adult_M2	1 μg	42268354	41848070	99.01	6 G
adult_M3	1 μg	50168748	49663416	98.99	6 G
adult_M4	1 μg	40891034	40460280	98.95	6 G
adult_M5	1 μg	48792070	48309322	99.01	6 G
adult_M6	1 μg	42208892	41761742	98.85	6 G

Table S2. Table of gut microbial genus and species level abundances of *O. furnacalis*.

Species	L1D2 (%)	L3D2 (%)	L5D2 (%)	Pupa (%)	Adult_F (%)	Adult_M (%)
Pseudomonas aeruginosa	14.026	20.535	24.941	12.081	22.571	20.577
Piscirickettsia salmonis	10.298	10.012	12.069	7.268	17.128	13.149
Acinetobacter baumannii	6.544	8.874	13.255	13.205	12.263	13.058
Wolbachia endosymbiont of Ceutorhynchus assimilis	3.795	3.837	5.382	2.323	4.698	6.020
Escherichia coli	1.628	1.335	2.104	11.837	2.237	5.037
Listeria welshimeri	3.143	3.220	4.683	1.386	3.798	3.089
Enterococcus mundtii	0.028	15.413	4.654	0.012	3.399	0.070
Aeromonas salmonicida	15.933	0.001	0.000	0.000	1.993	1.503
Enterococcus casseliflavus	0.012	7.728	2.007	1.452	0.046	0.038
Rickettsia endosymbiont of Ceutorhynchus assimilis	0.766	1.304	1.061	1.216	1.371	1.605
Stenotrophomonas maltophilia	5.775	0.006	0.025	0.537	0.556	0.474
Virgibacillus massiliensis	0.855	0.941	1.764	0.898	1.674	0.784
Klebsiella pneumoniae	0.730	0.831	1.310	0.838	1.443	1.176
Wolbachia endosymbiont of Psylliodes chrysocephala	0.634	1.024	1.326	1.071	0.901	1.350
Sphingobacteriaceae bacterium	0.631	0.961	1.240	0.748	0.996	1.265
Streptococcus pyogenes	1.130	0.686	0.901	0.778	1.782	0.690
Enterobacter cloacae complex sp. 2DZ2F20B	0.756	0.816	0.967	0.553	1.593	1.066
Kosakonia cowanii	0.004	0.004	0.010	6.323	0.009	0.003
Salmonella sp. hn-f5	0.131	0.065	0.293	5.246	0.102	0.062
Solemya velum gill symbiont	0.507	0.681	0.818	0.699	1.143	0.879
Candidatus Nephrothrix sp. EaCA	0.527	0.824	0.605	0.565	0.343	0.455
Macrococcus caseolyticus	0.417	0.421	0.428	0.515	0.566	1.097
Others	31.732	20.480	20.159	30.448	19.385	26.554
**Genus**						
Pseudomonas	31.623	16.722	22.717	10.439	24.396	21.514
Enterococcus	0.881	44.485	17.562	5.834	9.246	13.542
Acinetobacter	4.084	7.111	11.932	13.308	10.978	11.796
Piscirickettsia	6.378	7.959	10.930	6.444	15.259	11.572
Aeromonas	24.788	0.161	0.233	0.331	4.825	3.751
Wolbachia	2.902	4.225	6.538	3.083	5.322	7.014
Listeria	2.574	3.879	5.917	2.121	6.173	4.324
Escherichia	1.013	1.056	1.940	10.855	1.995	4.711
Stenotrophomonas	6.606	0.109	0.171	1.301	1.526	1.014
Enterobacter	0.751	1.204	2.176	3.495	1.806	1.129
Others	18.402	13.089	19.883	42.789	18.474	19.632

Table S3. Analysis of the differences of gut microbiome function at level 3 based on the KEGG database.

KEGG_L1	KEGG_L2	KEGG_L3	Ko
Environmental Information Processing	Signal transduction	Phosphatidylinositol signaling system	ko04070
Human Diseases	Endocrine and metabolic disease	Insulin resistance	ko04931
Organismal Systems	Digestive system	Fat digestion and absorption	ko04975
Environmental Information Processing	Signal transduction	Sphingolipid signaling pathway	ko04071
Organismal Systems	Nervous system	Neurotrophin signaling pathway	ko04722
Organismal Systems	Endocrine system	Insulin signaling pathway	ko04910
Cellular Processes	Cell growth and death	Apoptosis	ko04210
Human Diseases	Drug resistance: antineoplastic	Platinum drug resistance	ko01524
Organismal Systems	Development and regeneration	Axon guidance	ko04360
Environmental Information Processing	Signal transduction	mTOR signaling pathway	ko04150
Human Diseases	Neurodegenerative disease	Spinocerebellar ataxia	ko05017
Environmental Information Processing	Signal transduction	Rap1 signaling pathway	ko04015
Human Diseases	Infectious disease: viral	Human T-cell leukemia virus 1 infection	ko05166
Environmental Information Processing	Signal transduction	Ras signaling pathway	ko04014
Environmental Information Processing	Signal transduction	Hippo signaling pathway - fly	ko04391
Human Diseases	Cancer: overview	Viral carcinogenesis	ko05203
Human Diseases	Cancer: overview	MicroRNAs in cancer	ko05206
Metabolism	Carbohydrate metabolism	Citrate cycle (TCA cycle)	ko00020
Cellular Processes	Transport and catabolism	Peroxisome	ko04146
Organismal Systems	Sensory system	Phototransduction - fly	ko04745
